# Supplementary material for: Chemical Constituents of Thesium chinense Turcz and Their In Vitro Antioxidant, Anti-Inflammatory and Cytotoxic Activities
Source: Molecules. 2023 Mar 16;28(6):2685. doi: 10.3390/molecules28062685 (PMC10054634; doi:10.3390/molecules28062685)
Supplement: Supplementary file 1 [file molecules-28-02685-s001.zip › molecules-2238612-SI.pdf]

SUPPLEMENTARY MATERIAL

Chemical constituents of *Thesium chinense* Turcz and their in vitro antioxidant, anti-inflammatory and cytotoxic activities

Zhen-Zhen Liu, Jun-Cheng Ma, Peng Deng, Fu-Cai Ren*, Ning Li*

Inflammation and Immune Mediated Diseases Laboratory of Anhui Province, School of Pharmacy, Anhui Medical University, Hefei, 230032, China

Content

Fig. S1. The ^{1}H NMR spectrum of compound **1**. 1

Fig. S2. The ^{3}C-DEPT-135 spectrum of compound **1** 1

Fig. S3. The ^{13}C NMR spectrum of compound **1** 1

Fig. S4. The HSQC spectrum of compound **1**. 2

Fig. S5. The HMBC spectrum of compound **1** 2

Fig. S6. ^{1}H-^{1}H COSY spectrum of compound **1** 3

Fig. S7. The NOESY spectrum of compound **1** 3

Fig. S8. The MS-ESI spectrum of compound **1** 4

Fig. S9. The HRMS-ESI spectrum of compound **1** 4

Fig. S10. The IR spectrum of compound **1** 5

Fig. S11. The UV spectrum of compound **1**. 5

Fig. S12. The rotation spectrum of compound **1** 5

Table S1. “GIAO” method results of **1a** and **1b** 6

Table S2. Energies of the calculated configuration of **1a**. 6

Table S3. Energies of the calculated configuration of **1b** 7

Fig. S13. The ^{1}H NMR spectrum of compound **2**. 8

Fig. S14. The ^{3}C-DEPT-135 spectrum of compound **2** 8

Fig. S15. The ^{13}C NMR spectrum of compound **2** 8

Fig. S16. The HSQC spectrum of compound **2**. 9

Fig. S17. The HMBC spectrum of compound **2** 9

Fig. S18. ^{1}H-^{1}H COSY spectrum of compound **2** 10
Fig. S19. The MS-ESI spectrum of compound **2**. 11
Fig. S20. The HRMS-ESI spectrum of compound **2** 11
Fig. S21. The IR spectrum of compound **2** 12
Fig. S22. The UV spectrum of compound **2**. 12
Fig. S23. The rotation spectrum of compound **2** 12
Fig. S24. The ^{1}H NMR spectrum of compound **3**. 13
Fig. S25. The ^{3}C-DEPT-135 spectrum of compound **3** 13
Fig. S26. The ^{13}C NMR spectrum of compound **3** 13
Fig. S27. The HSQC spectrum of compound **3**. 14
Fig. S28. The HMBC spectrum of compound **3** 14
Fig. S29. ^{1}H-^{1}H COSY spectrum of compound **3** 15
Fig. S30. The MS-ESI spectrum of compound **3**. 16
Fig. S31. The HRMS-ESI spectrum of compound **3** 16
Fig. S32. The IR spectrum of compound **3** 17
Fig. S33. The UV spectrum of compound **3**. 17
Fig. S34. The rotation spectrum of compound **3** 17

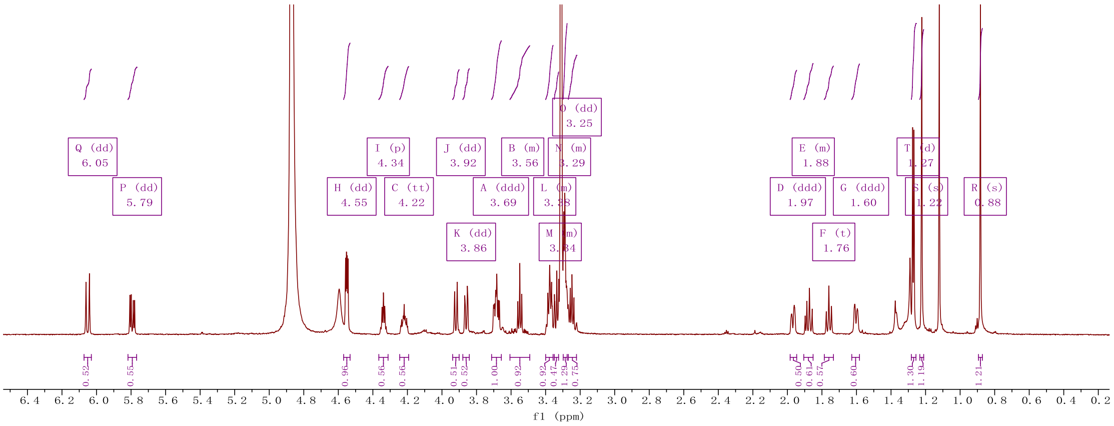

Fig. S1. The ^{1}HNMR spectrum (800 MHz, CD_3OD) of compound **1**.

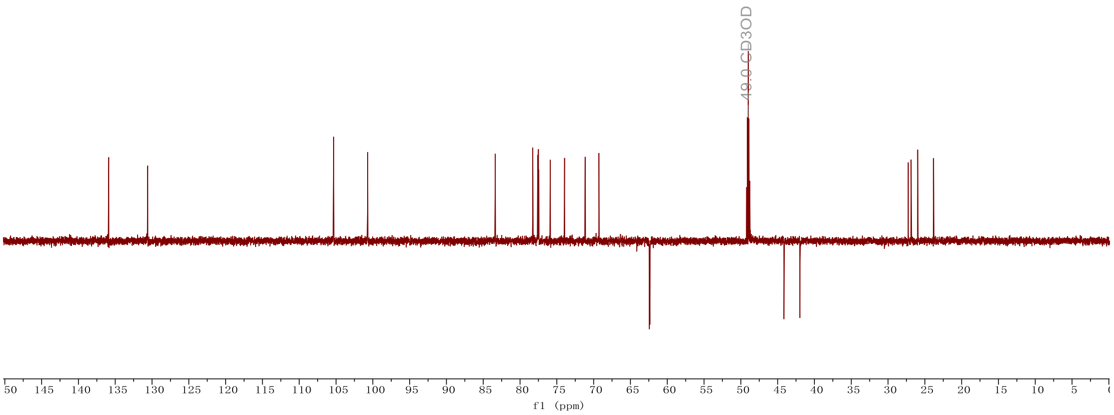

Fig. S2. The ^{13}C-DEPT-135 spectrum (2000 MHz, CD_3OD) of compound **1**.

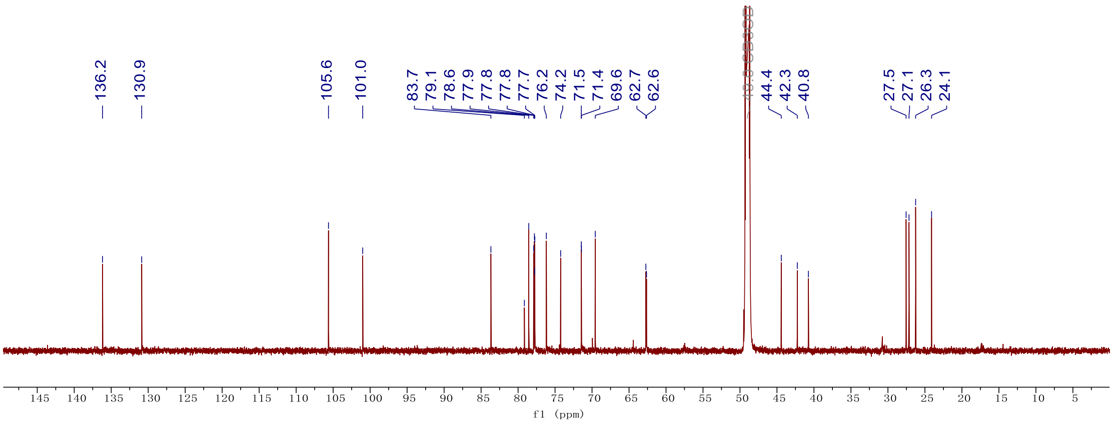

Fig. S3. The ^{13}C NMR spectrum (200 MHz, CD_3OD) of compound **1.**

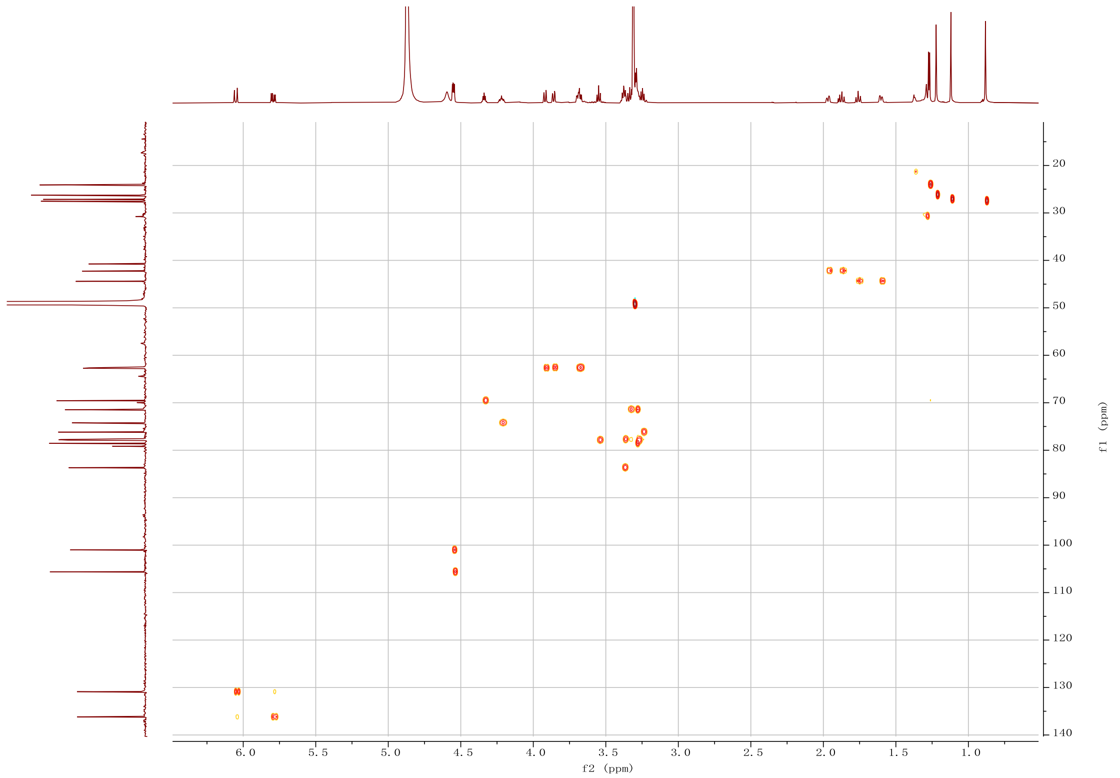

Fig. S4. The HSQC spectrum of compound **1**.

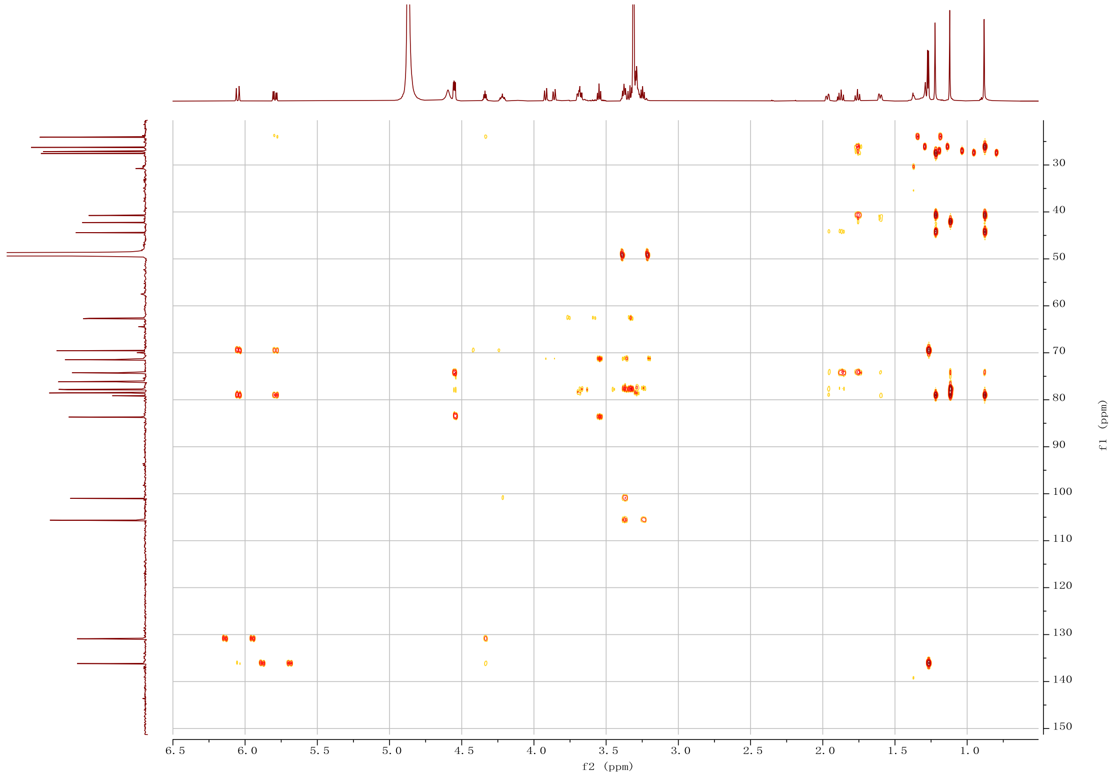

Fig. S5. The HMBC spectrum of compound **1**.

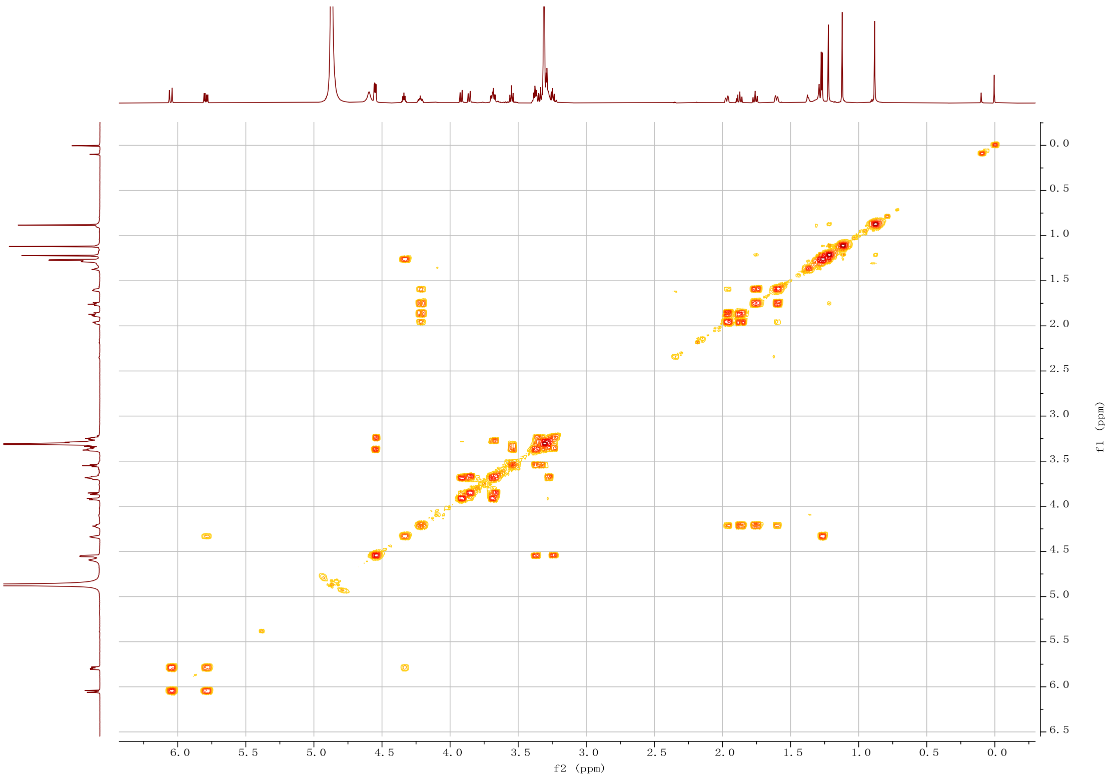

Fig. S6. ^{1}H-^{1}H COSY spectrum of compound **1**.

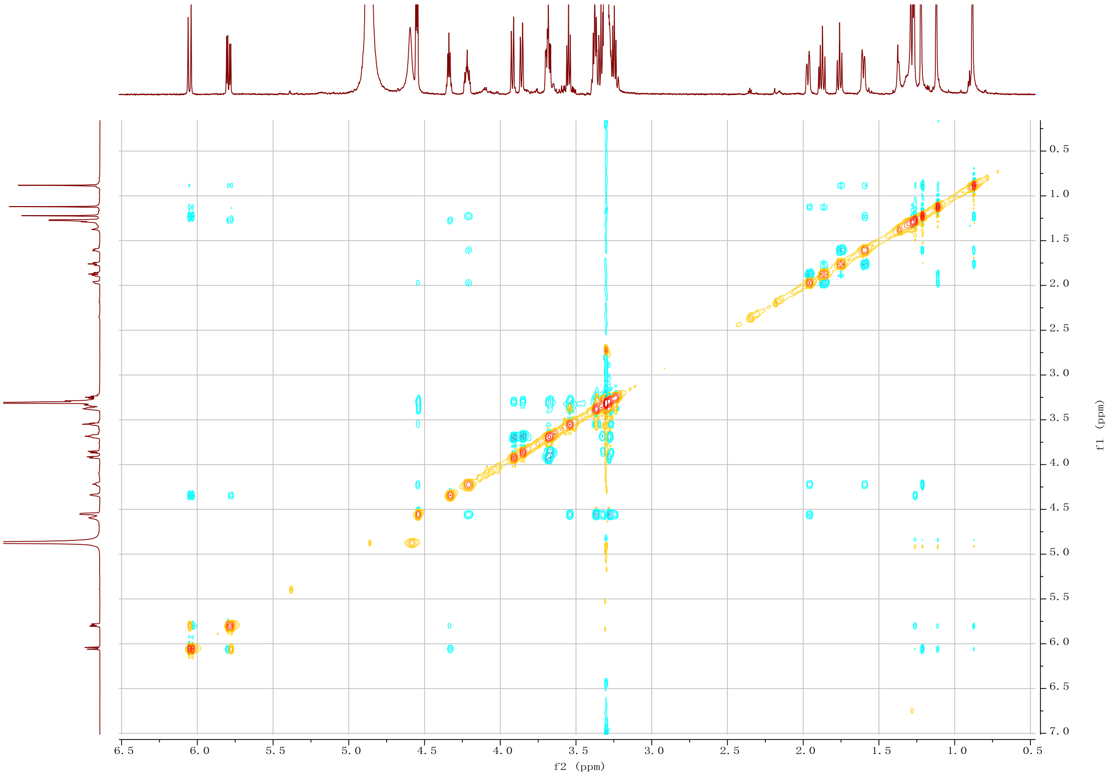

Fig. S7. The NOESY spectrum of compound **1**.

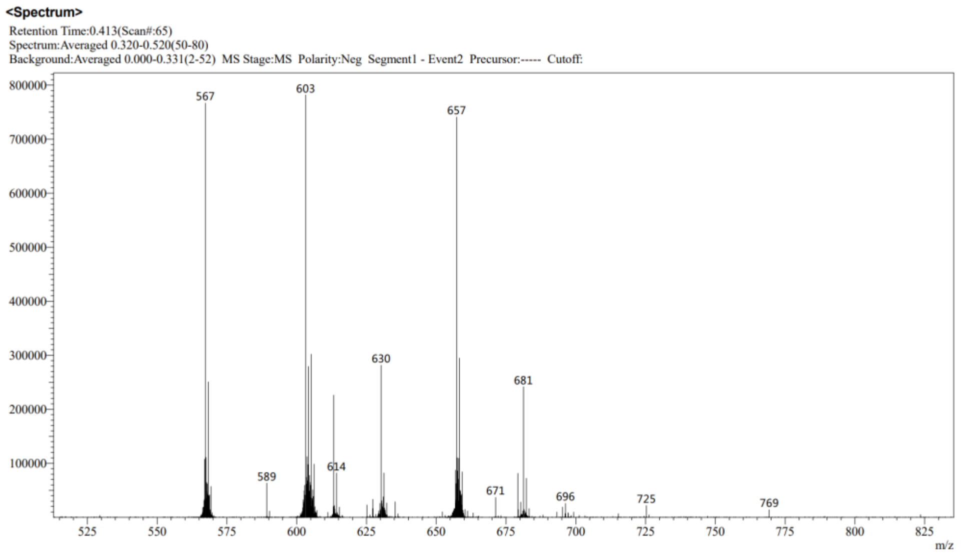

Fig. S8. MS-ESI (−) spectrum of compound **1**.

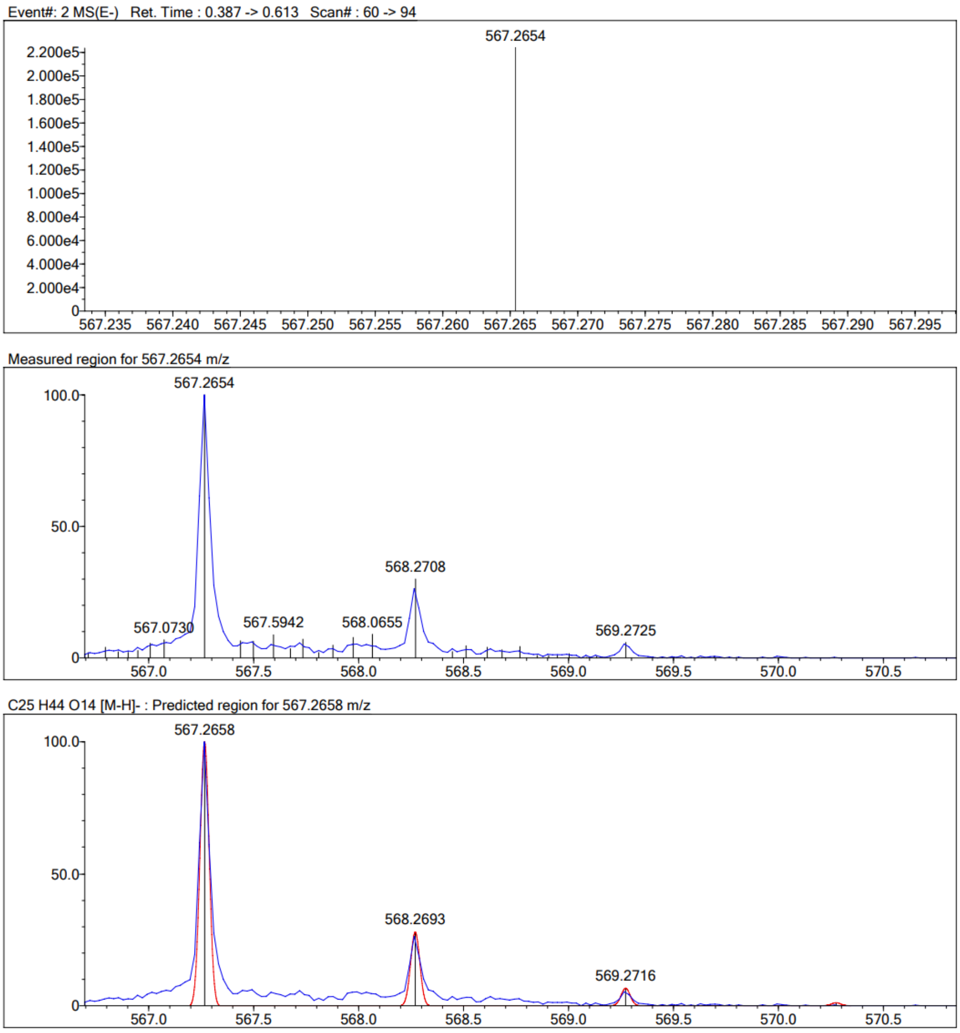

Formula (M)	Ion	Meas. m/z	Pred. m/z	Df. (mDa)	Df. (ppm)	DBE
C25 H44 O14	[M-H]-	567.2654	567.2658	-0.4	-0.71	4.0

Fig. S9. HRMS-ESI (−) spectrum of compound **1**.

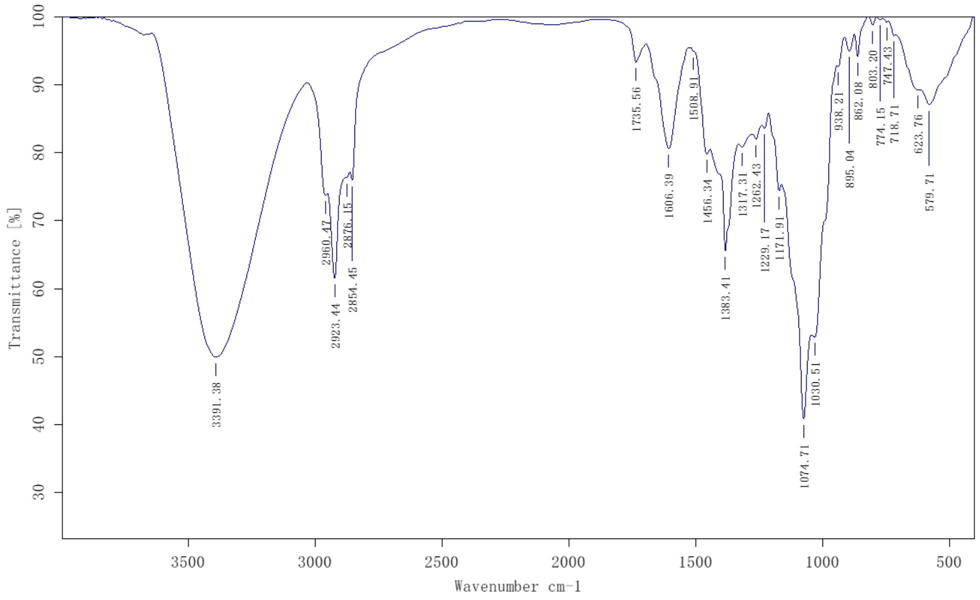

Fig. S10. IR spectrum of compound **1**.

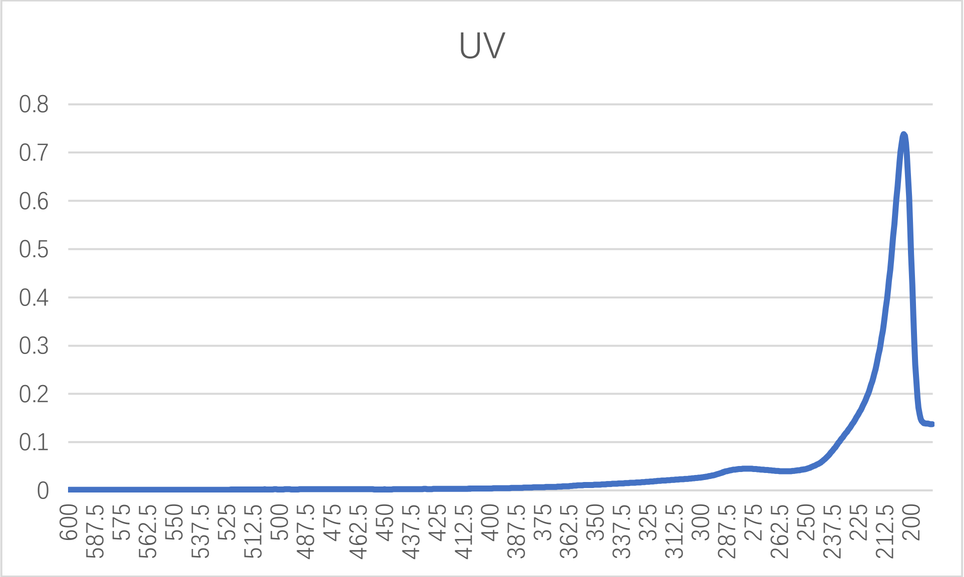

Fig. S11. UV spectrum of compound **1**.

Rudolph Research Analytical

This sample was measured on an Autopol VI, Serial #91058
Manufactured by Rudolph Research Analytical, Hackettstown, NJ, USA.

Measurement Date : Friday, 22-JUL-2022

Set Temperature : OFF

Time Delay : Disabled

Delay between Measurement : Disabled

n	Average	Std.Dev.	% RSD	Maximum	Minimum
5	-26.70	0.23	-0.86	-26.50	-27.10

S.No	Sample ID	Time	Result	Scale	OR °Arc	WLG.nm	Lg.mm	Conc.g/100ml	Temp.
1	ZL-3	01:59:44 PM	-27.10	SR	-0.0271	589	100.00	0.100	25.4
2	ZL-3	01:59:52 PM	-26.70	SR	-0.0267	589	100.00	0.100	25.5
3	ZL-3	02:00:01 PM	-26.60	SR	-0.0266	589	100.00	0.100	25.4
4	ZL-3	02:00:09 PM	-26.50	SR	-0.0265	589	100.00	0.100	25.4
5	ZL-3	02:00:17 PM	-26.60	SR	-0.0266	589	100.00	0.100	25.4

Fig. S12. Rotation spectrum of compound **1**.

Table S1 Statistics of Ordinary Least Squares (OLS) Linear Regression of experimental and computed ^{13}C-NMR chemical shifts.

Configuration	DP4+ probability (%)	R^2	RMSE
**1a**	99.96	0.9904	3.13
**1b**	0.04	0.9861	3.63

Energies at M062X/6-31G(d) theory level

Energies of the calculated configuration of **1a.**

Table S2 Energies and populations of conformers of the calculated configuration.

Conformer	Population（%）	E（Hartree）
**1**	1.47	-2031.385069
**6**	44.86	-2031.388295
**12**	5.14	-2031.386251
**18**	45.94	-2031.388317
**19**	1.35	-2031.384992
**21**	1.24	-2031.384912

Energies of the calculated configuration of **1b.**

Table S3 Energies and populations of conformers of the calculated configuration.

Conformer	Population（%）	E（Hartree）
**1**	3.87	-2031.387785
**2**	5.2	-2031.388062
**3**	5.49	-2031.388113
**4**	6.49	-2031.388271
**5**	3.18	-2031.3876
**6**	4.53	-2031.387933
**7**	7.1	-2031.388357
**8**	3.44	-2031.387673
**9**	1.13	-2031.386623
**10**	1.55	-2031.386921
**11**	2.17	-2031.387239
**12**	4.15	-2031.387849
**17**	1.24	-2031.386711
**20**	3.05	-2031.387559
**22**	1.18	-2031.386662
**24**	5.55	-2031.388125
**25**	4.84	-2031.387995
**53**	1.58	-2031.386941
**62**	1.98	-2031.387152
**66**	2	-2031.387161
**90**	3.15	-2031.38759
**92**	4.61	-2031.387949
**94**	3.87	-2031.387784
**116**	5.94	-2031.388188
**120**	5.65	-2031.388141
**122**	7.07	-2031.388352

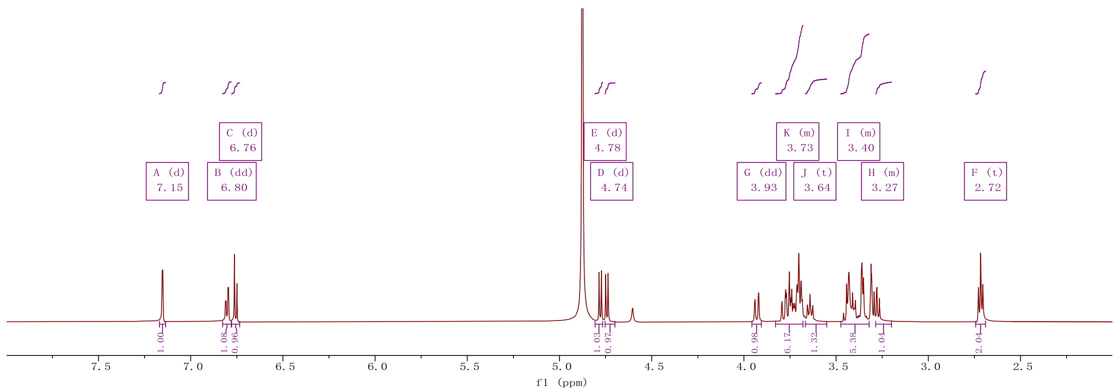

Fig. S13. The ^{1}HNMR spectrum (600 MHz, CD_3OD) of compound **2**.

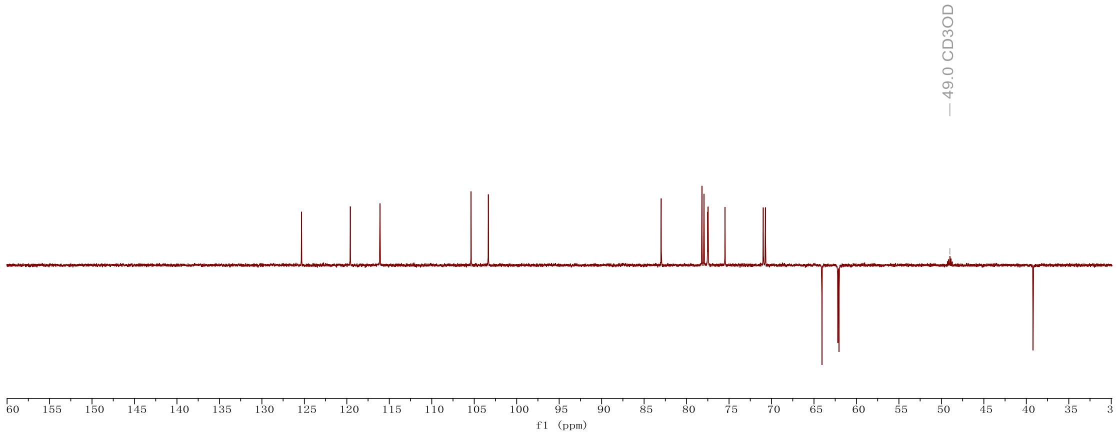

Fig. S14. The ^{13}C-DEPT-135 spectrum (150 MHz, CD_3OD) of compound **2**.

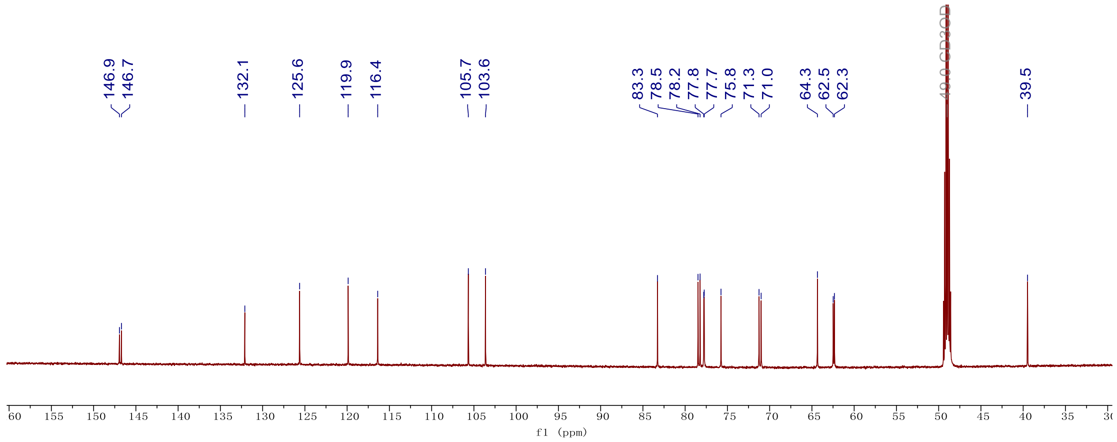

Fig. S15. The ^{13}C NMR spectrum (150 MHz, CD_3OD) of compound **2**.

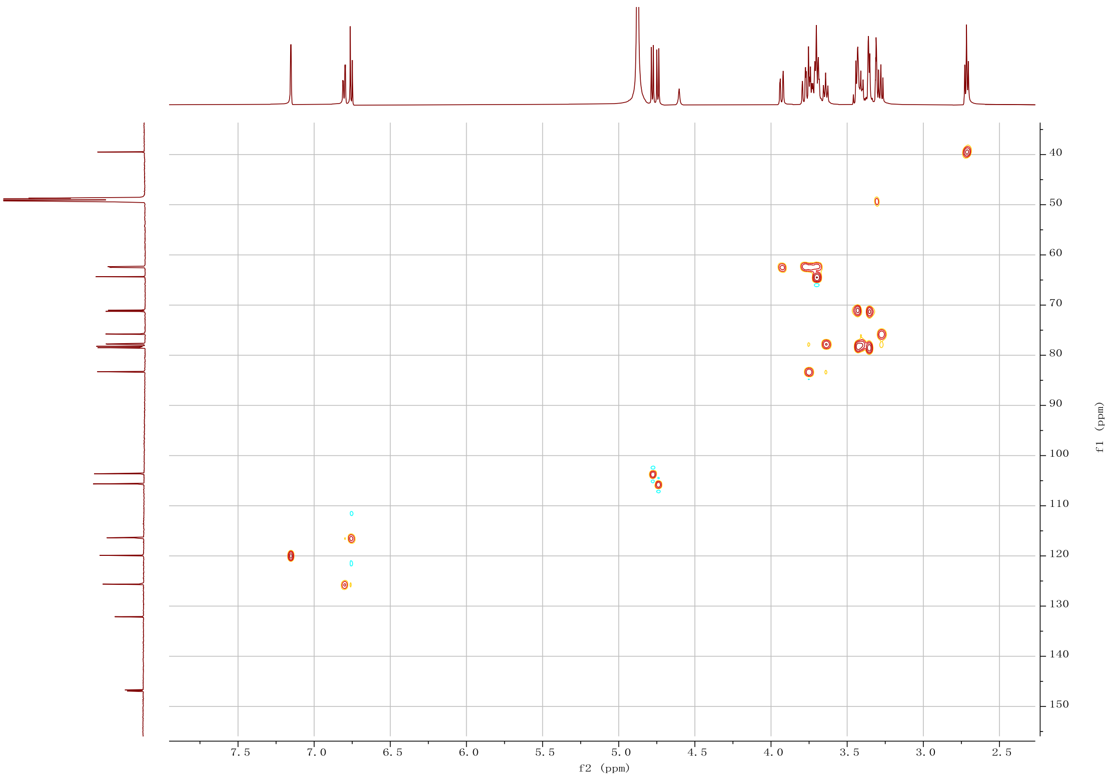

Fig. S16. The HSQC spectrum of compound **2**.

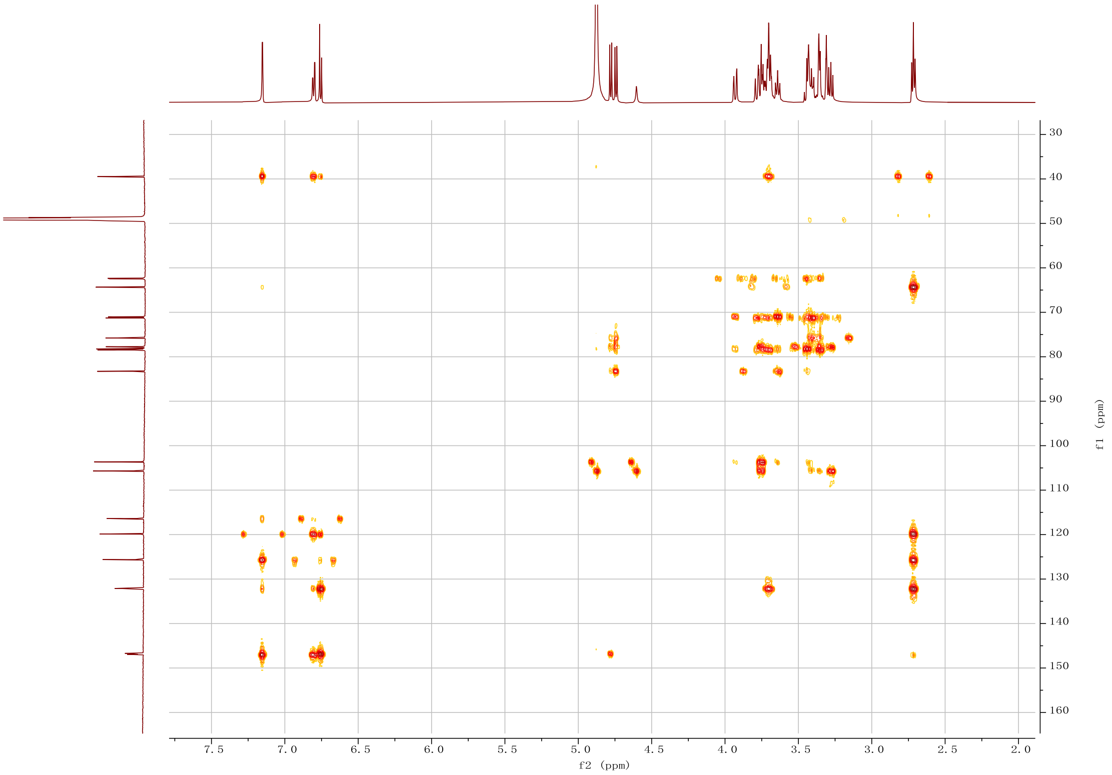

Fig. S17. The HMBC spectrum of compound **2**.

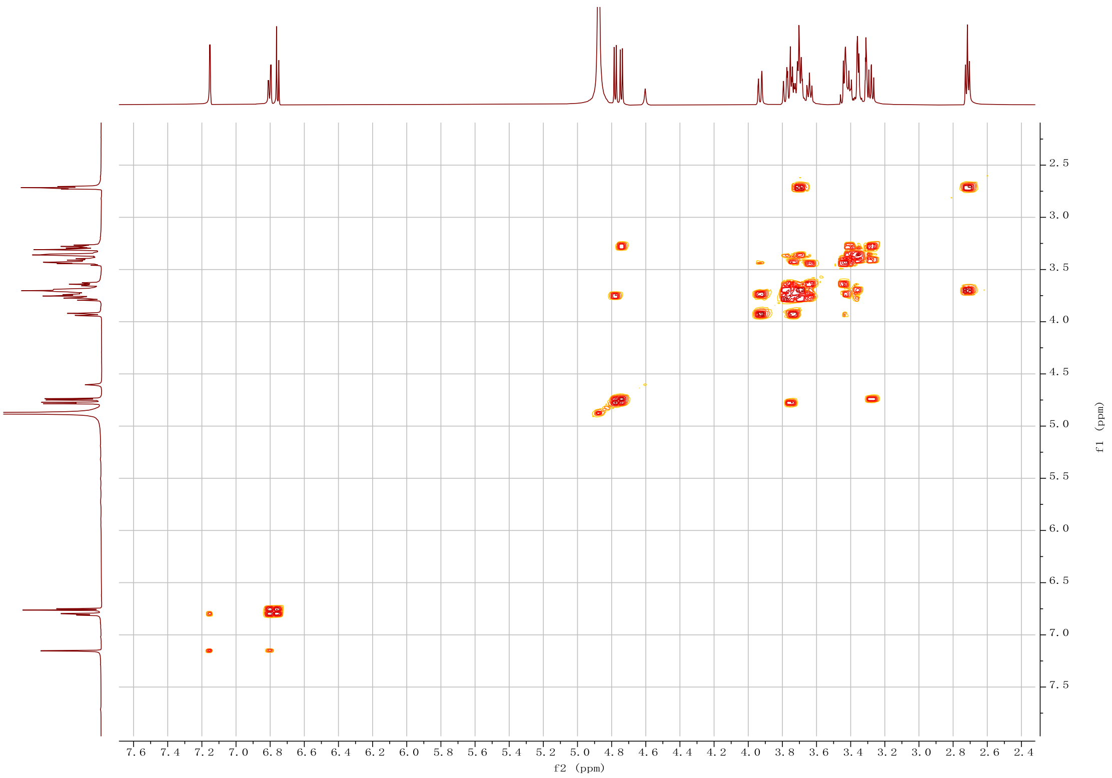

Fig. S18. ^{1}H-^{1}H COSY spectrum of compound **2**.

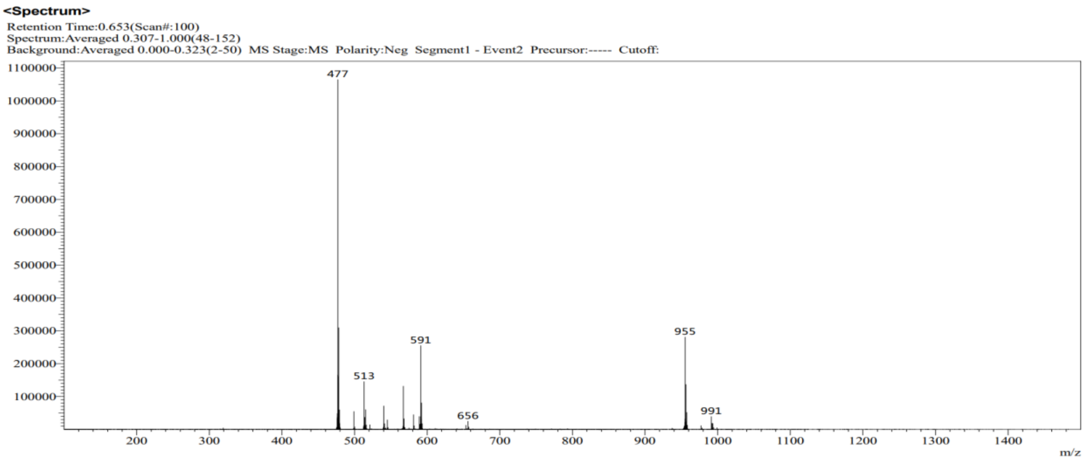

Fig. S19. MS-ESI (−) spectrum of compound **2**.

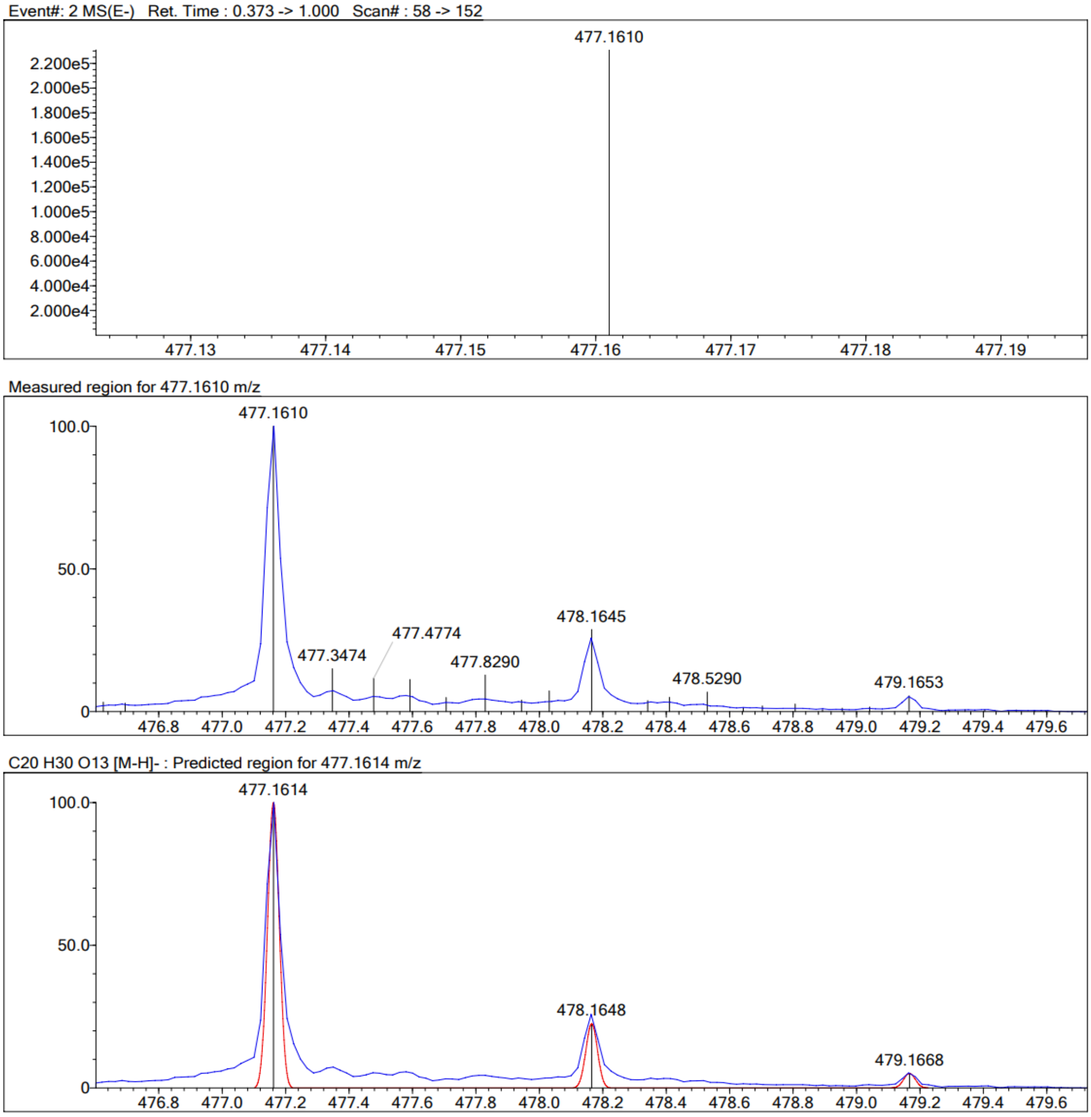

Formula (M)	Ion	Meas. m/z	Pred. m/z	Df. (mDa)	Df. (ppm)	DBE
C20 H30 O13	[M-H]-	477.1610	477.1614	-0.4	-0.84	6.0

Fig. S20. HRMS-ESI (−) spectrum of compound **2**.

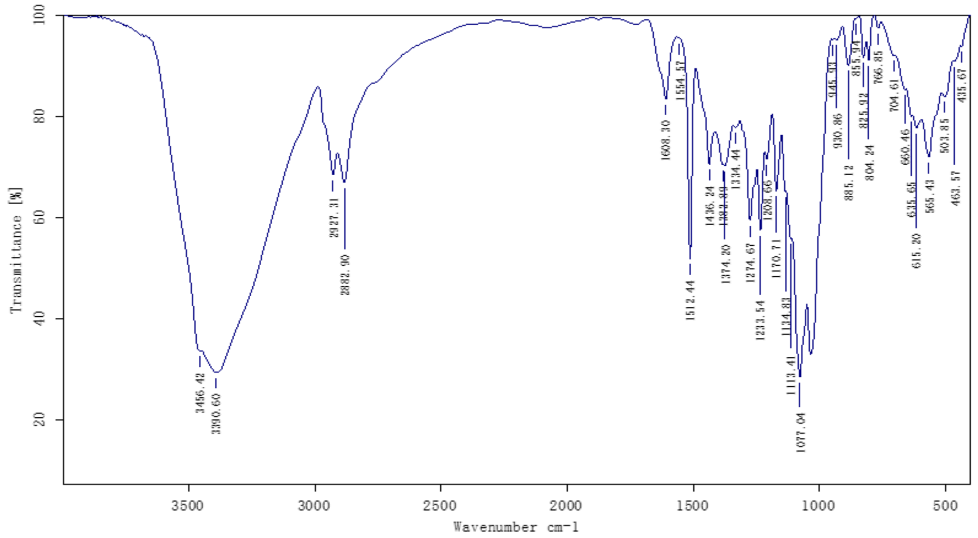

Fig. S21. IR spectrum of compound **2**.

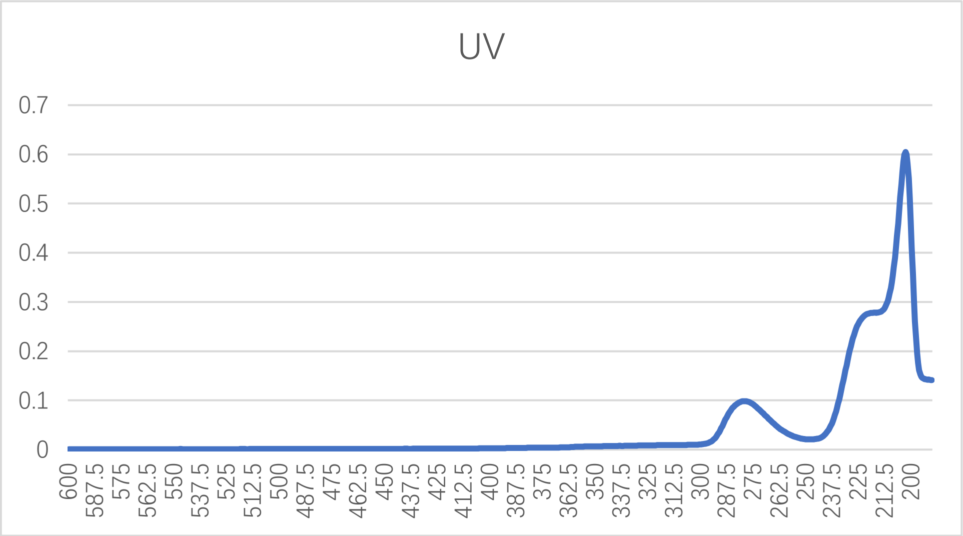

Fig. S22. UV spectrum of compound **2**.

Rudolph Research Analytical

This sample was measured on an Autopol VI, Serial #91058
Manufactured by Rudolph Research Analytical, Hackettstown, NJ, USA.

Measurement Date : Friday, 22-JUL-2022

Set Temperature : OFF

Time Delay : Disabled

Delay between Measurement : Disabled

n	Average	Std.Dev.	% RSD	Maximum	Minimum
5	-1.20	0.10	-8.33	-1.07	-1.27

S.No	Sample ID	Time	Result	Scale	OR °Arc	WLG.nm	Lg.mm	Conc.g/100ml	Temp.
1	ZL-5	02:06:13 PM	-1.27	SR	-0.0038	589	100.00	0.300	25.5
2	ZL-5	02:06:21 PM	-1.27	SR	-0.0038	589	100.00	0.300	25.5
3	ZL-5	02:06:30 PM	-1.27	SR	-0.0038	589	100.00	0.300	25.5
4	ZL-5	02:06:38 PM	-1.07	SR	-0.0032	589	100.00	0.300	25.5
5	ZL-5	02:06:46 PM	-1.10	SR	-0.0033	589	100.00	0.300	25.5

Fig. S23. Rotation spectrum of compound **2**.

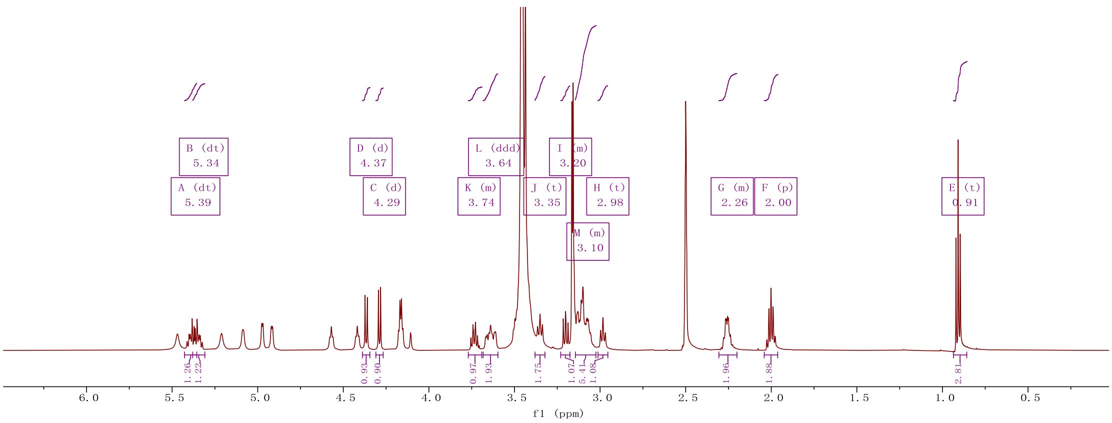

Fig. S24. The ^{1}HNMR spectrum (600 MHz, CD_3OD) of compound **3**.

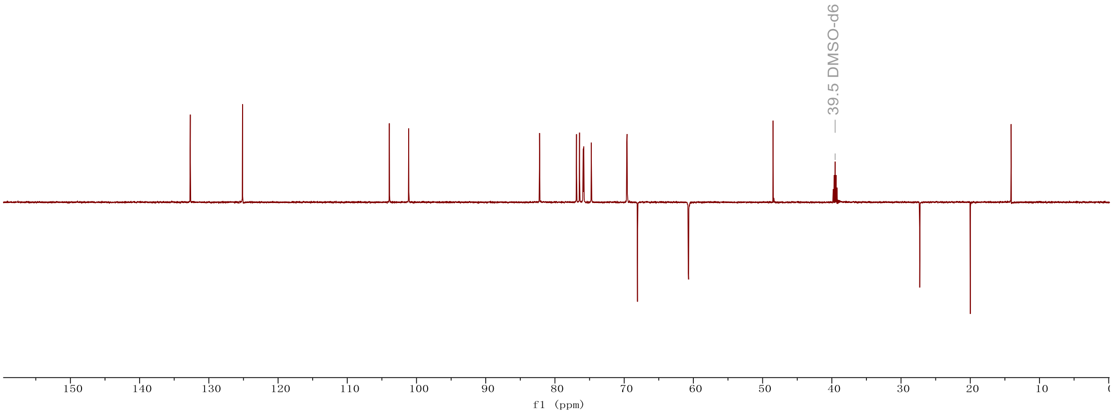

Fig. S25. The ^{13}C-DEPT-135 spectrum (150 MHz, CD_3OD) of compound **3**.

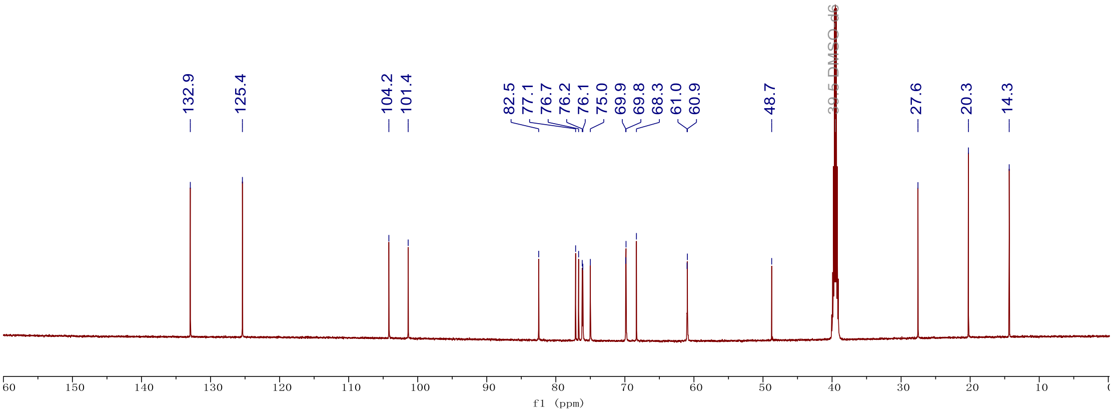

Fig. S26. The ^{13}C NMR spectrum (150 MHz, CD_3OD) of compound **3.**

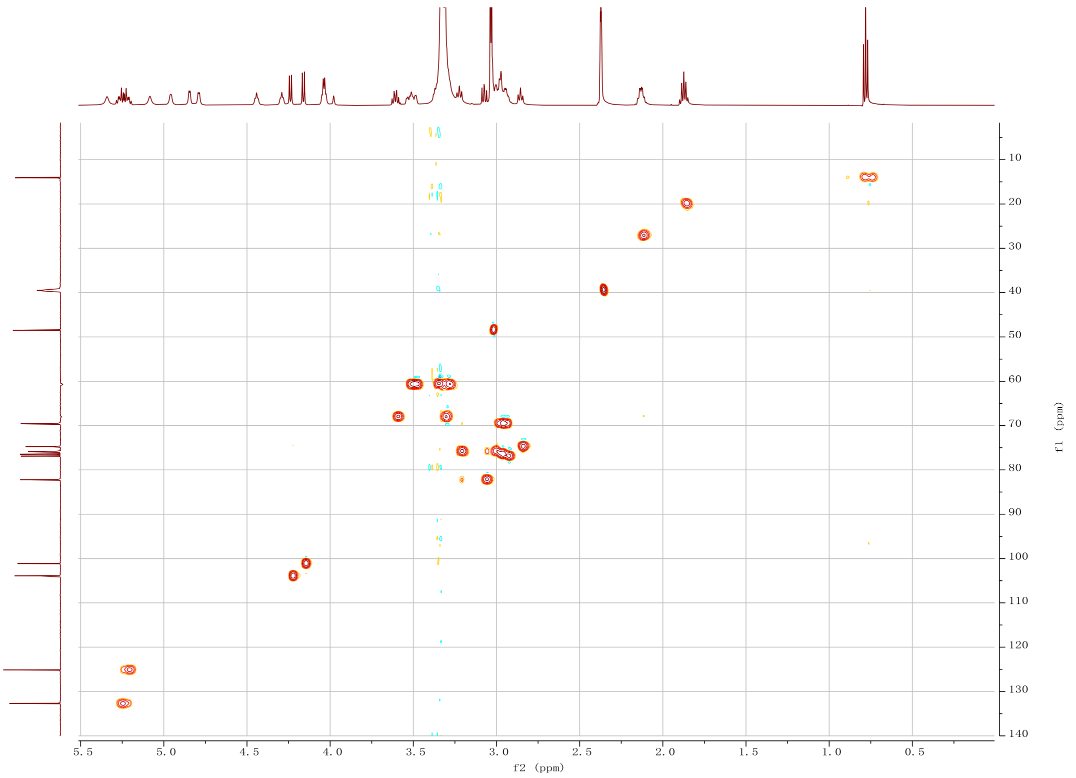

Fig. S27 The HSQC spectrum of compound **3**.

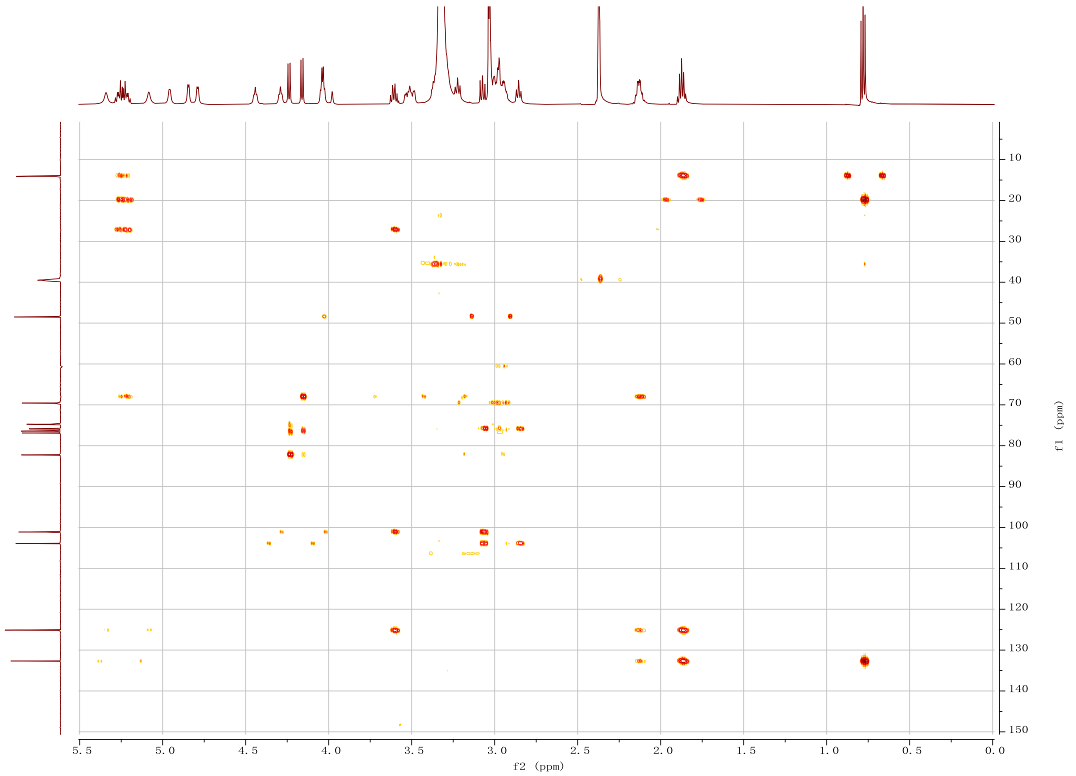

Fig. S28. The HMBC spectrum of compound **3**.

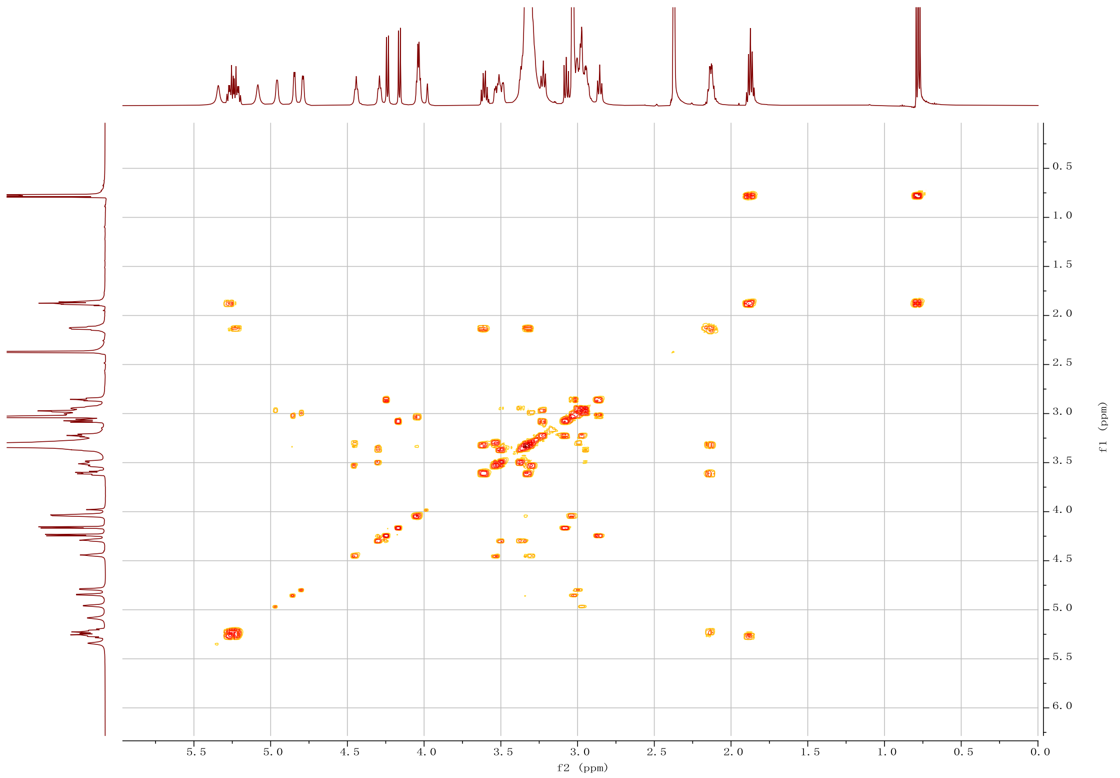

Fig. S29. ^{1}H-^{1}H COSY spectrum of compound **3**.

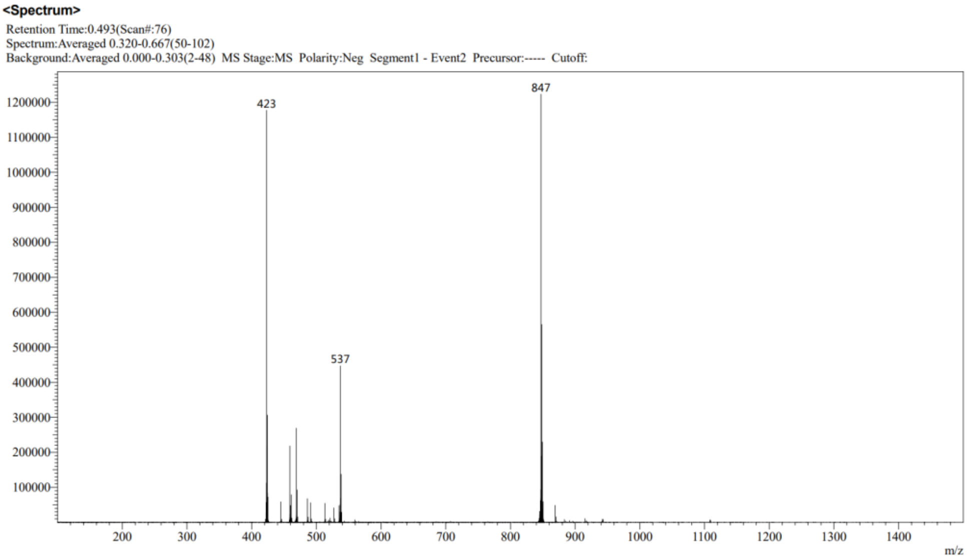

Fig. S30. MS-ESI (−) spectrum of compound **3**.

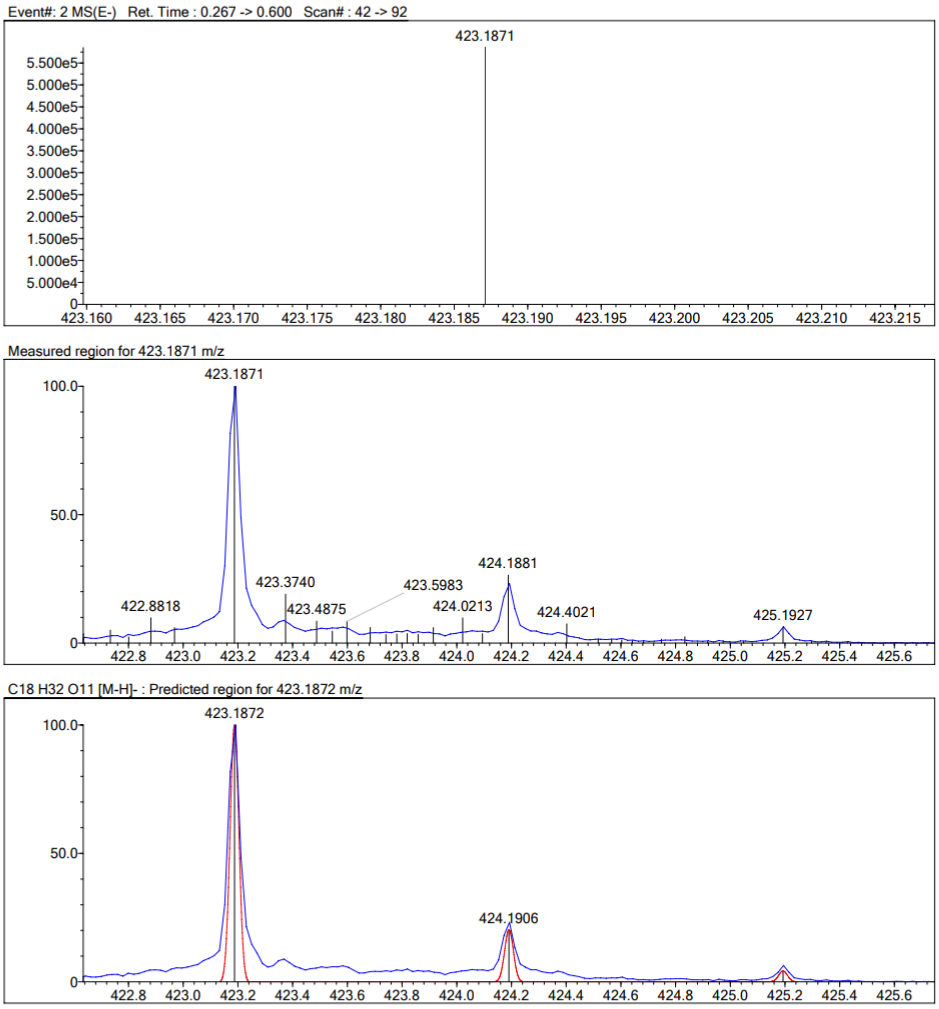

Formula (M)	Ion	Meas. m/z	Pred. m/z	Df. (mDa)	Df. (ppm)	DBE
C18 H32 O11	[M-H]-	423.1871	423.1872	-0.1	-0.24	3.0

Fig. S31. HRMS-ESI (−) spectrum of compound **3**.

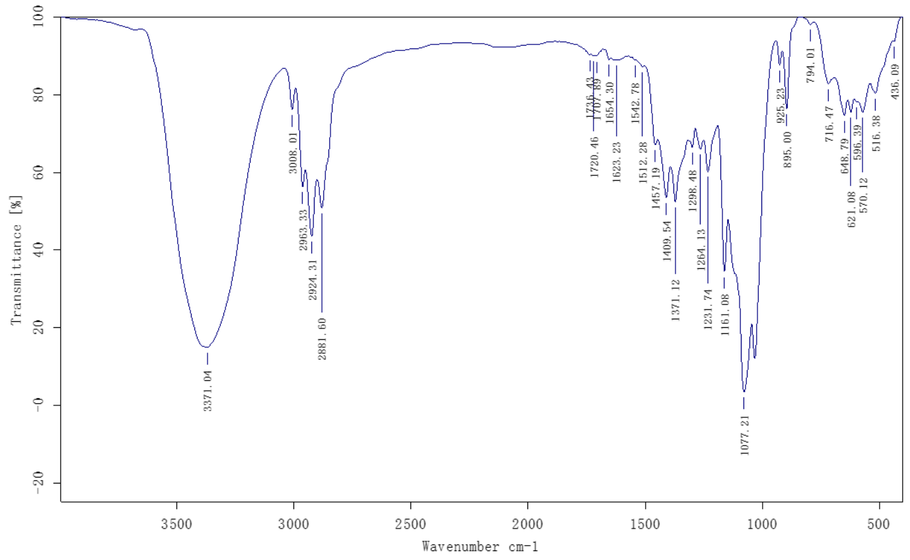

Fig..S32. IR spectrum of compound **3**.

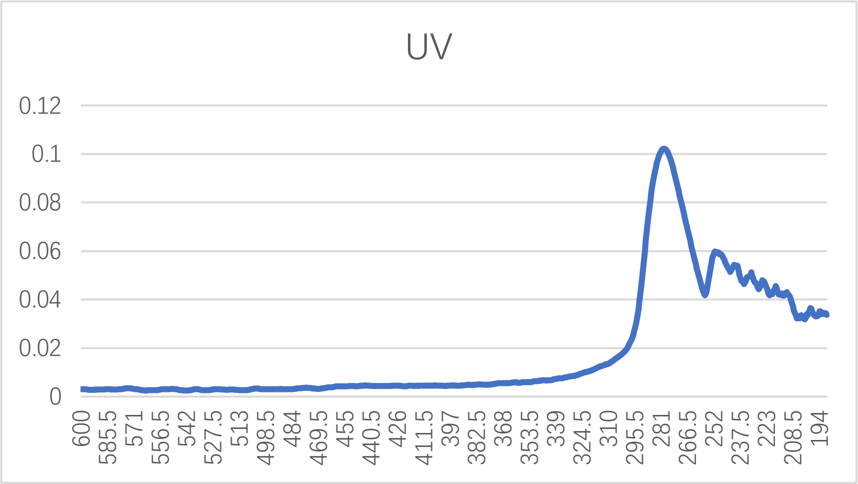

Fig. S33. UV spectrum of compound **3**.

Rudolph Research Analytical

This sample was measured on an Autopol VI, Serial #91058
Manufactured by Rudolph Research Analytical, Hackettstown, NJ, USA.

Measurement Date : Friday, 22-JUL-2022

Set Temperature : OFF

Time Delay : Disabled

Delay between Measurement : Disabled

n	Average	Std.Dev.	% RSD	Maximum	Minimum
5	-14.22	0.13	-0.91	-14.06	-14.36

S.No	Sample ID	Time	Result	Scale	OR °Arc	WLG.nm	Lg.mm	Conc.g/100ml	Temp.
1	ZL-2	01:28:22 PM	-14.06	SR	-0.1406	589	100.00	1.000	24.6
2	ZL-2	01:28:30 PM	-14.13	SR	-0.1413	589	100.00	1.000	24.6
3	ZL-2	01:28:38 PM	-14.36	SR	-0.1436	589	100.00	1.000	24.6
4	ZL-2	01:28:55 PM	-14.33	SR	-0.1433	589	100.00	1.000	24.6
5	ZL-2	01:29:04 PM	-14.21	SR	-0.1421	589	100.00	1.000	24.7

Fig. S34. Rotation spectrum of compound **3**.
